# Implementing a Team-Based Fourth-Year Medical Student Rotation in Emergency Medicine

**DOI:** 10.5811/westjem.2017.11.36511

**Published:** 2017-12-18

**Authors:** Matthew Tews, Robert W. Treat

**Affiliations:** * Medical College of Georgia, Department of Emergency Medicine, Augusta, Georgia; † Medical College of Wisconsin, Department of Emergency Medicine, Milwaukee, Wisconsin

## BACKGROUND

Historically, fourth-year (M4) medical students at the Medical College of Wisconsin who were interested in emergency medicine (EM) worked with faculty in the emergency department primarily in a see-one-staff-one clinical model. Students were sent to see a patient, obtain a history and perform a physical examination and then present a summary to the faculty.^1^,^2^,^3^ The faculty evaluations of these student interactions constituted the majority of the student’s clinical score and clerkship grade.^4^ However, in the busy clinical environment of a Level I trauma center, it was challenging for faculty to dedicate one-on-one time with students. As a result, students frequently waited on busy faculty to assign them a new patient rather than proactively gaining clinical experience. Therefore, a new team-based clinical model was created after obtaining input from faculty, residents and students to increase student interactions with patients. Although similar models might exist at other programs, there was limited evidence to guide a student’s team function in the ED.^5^,^6^ As a result, it was decided to study the impact of the change on student evaluations and case logs, as well as faculty and student perceptions, all of which stood to be impacted the most by the change.

## OBJECTIVES

The objectives for this educational innovation were to 1) implement a team-based model of a M4 student clinical experience; 2) measure the student’s clinical performance from their end-of-shift evaluations and case logs; and 3) assess the perception of the model from faculty and students.

## CURRICULAR DESIGN

We obtained a prospective collection of data from 32 M4 students over a four-month time period from July to October 2015. Students were randomly assigned to a geographic team that used either the team-based model or traditional staffing model. Faculty and residents received instructions on expectations in the months prior to implementation via emails, group presentations and face-to-face meetings, while students were informed at each month’s orientation. Students in the traditional model were assigned to faculty without a change in expectations compared to previous years. In the team-based model, students continued to work one-on-one with faculty when convenient, but they were also expected to proactively contribute to the care of any patient on their team or when asked by the faculty or resident.

Examples of student contributions included helping with procedures, calling the poison center, gathering history from nursing homes and interacting with consultants for any team patient. Faculty completed end-of-shift evaluations in both models; faculty and students completed a survey regarding their experiences. We analyzed student case logs to determine their level of involvement with faculty (Table). Narrative comments were analyzed to understand faculty and student perceptions. We conducted analysis by IBM® SPSS® v21.0. The study was deemed exempt by the Medical College of Wisconsin Institutional Review Board.

## IMPACT/EFFECTIVENESS

Our preliminary work suggested that the team-based model provided students more involvement in patient care without negatively impacting their clinical evaluations. Of the 339 end-of-shift evaluations completed by faculty, there were no statistically significant differences in how faculty assessed students between the two models; however, the team-based model reported a trend towards higher mean scores. Students saw significantly more patients in the team-based model (p=.000) with 58% of patients seen as part of the team, while students in the traditional model saw 60% of patients one-on-one with faculty (Table). This demonstrated that the increase in patient involvement for the team-based model occurred when students participated as part of the team.

In reviewing the faculty and students’ perceptions, we found that many students saw the value in this model, commenting that “my skills and knowledge could be utilized and [I could] function as a valuable member of the team” and that it “allowed me to see more patients, be helpful to the team, learn more and prepare to handle multiple patients.” Some students preferred the traditional model because it allowed “time for informed decision-making,” and it “felt like we were pulled in less directions.” Faculty viewed this as an opportunity to help differentiate students, commenting that the model “pushes students to be proactive and helps separate out students a bit more who took initiative versus who didn’t.” Students highly rated their interactions with faculty and the faculty’s excellence in teaching in both models.

Limitations of this study include that the case logs were self-reported by students and that faculty evaluations of students are subjective measures of student performance, despite use of a standardized form. Additionally, in retrospect we found that while the deliberate rollout of the model helped socialize the idea, more time could have been spent post-implementation verifying that the model was understood and adopted correctly by faculty, residents and students. This would have likely facilitated a quicker transition, although the model successfully became part of the department’s educational culture.

## Figures and Tables

**Table:** Student level of involvement in EM clerkship, comparing team-based vs. traditional clinical involvement.

	Team-based		Traditional	
		
Level of Involvement	Number of patients	Percent of total patients	Number of patients	Percent of total patients
Primarily saw patient with faculty only	589	32.5%	686	63.1%
Saw patient in team-based model; followed majority of time	583	32.2%	110	10.1%
Saw patient in team-based model, with minor involvement	459	25.3%	99	9.1%
Shadow faculty member only	182	10.0%	192	17.7%
Total	1911	100.0%	1135	100.0%
